# Eutectic Thin-Layer Chromatography as a New Possibility for Quantification of Plant Extracts—A Case Study

**DOI:** 10.3390/molecules27092960

**Published:** 2022-05-05

**Authors:** Danuta Raj

**Affiliations:** Department of Pharmacognosy and Herbal Medicines, Wroclaw Medical University, Borowska 211a, 50-556 Wrocław, Poland; danuta.raj@umw.edu.pl

**Keywords:** alkaloids, *Chelidonium*, densitometry, DES, eutectic TLC, NADES

## Abstract

Deep eutectic solvents (DES), compared to classic ones, have interesting properties, such as the ability to solubilize compounds differing in polarity or increased dissolution of selected chemical compounds. They also offer specific interactions between the mobile and stationary phases. Those features make them promising solvents in chromatographic techniques, including the use in the separation of complicated samples. The first quantitative analysis with eutectic thin-layer chromatography (TLC) is presented in the paper. As a case study, five alkaloids from *Chelidonium maius* were selected as target compounds. A wide range of terpene-based DESs was investigated to develop the chromatographic system, both pure and after dilution. Moreover, a novel approach was employed to adjust polarity, involving mixing DESs differing in chromatographic properties. This procedure has proved to be effective. The best results were obtained with a 2:1 (wt/wt) mixture of DESs: camphor + phenol and menthol + limonene, with a 20% addition of methanol. The chromatographic system was validated and checked on the real sample, which made it the first applicable and operational quantitative eutectic TLC system.

## 1. Introduction

The first noted observation of eutexia dates back to the mid-16th c. and regards the classic cooling mixture composed of ice and salt [1]. However, it was Isaac Newton who first correctly identified the eutexia phenomenon as the lowering of the mixture’s freezing point compared to the individual compounds, and in 1701, he created a low-melting alloy [2]. However, it took as much as three centuries for life-science researchers to recognize and start realizing the significance of the occurrence of eutectic mixtures in life sciences. Natural deep eutectic solvents (NADES) are the long-missing-link in understanding, for example, frost resistance, the ability to survive cryopreservation [3] or cell functioning in terms of compounds and processes that are incompatible with both hydrophobic and hydrophilic cell environments [4,5]. The ability to form eutectic mixtures by a wide range of natural compounds, including sugars, amino acids, organic acids, phenolic compounds or terpenes, undermines the previous strict division into primary and secondary metabolites and their hitherto presumed role in a living organism [4,5] and forces the life-science researchers to change the very bases of their view on the importance of specific compounds for the cell during its life cycle.

Deep eutectic solvents (DES) are being recently employed in multiple analytical applications that have been comprehensively reviewed in numerous papers [6,7,8,9]. From the very first reports about NADES [10,11], their usefulness as extraction media has been emphasized, especially regarding polyphenolics. DESs are also being used in separation processes [12,13,14,15]. The main reasons for which eutectic solvents are of such interest are their unique properties, which allow, among others, increased dissolution of selected compounds compared to classic organic solvents [10], the ability to solubilize compounds of a different polarity [16], and the possibility of fine-tuning DES’s polarity according to the needs [13,15]. Those features make eutectic liquids the prospective chromatographic solvents, allowing for novel solutions to the existing chromatographic difficulties.

This paper is a continuation of the previously published work [13], in which the preliminary results of the eutectic thin-layer chromatography (TLC) were described. As a case study, five alkaloids from *Chelidonium maius* were selected as target compounds. According to the postulates formed in the previous article, the validated eutectic TLC system is presented here for the first time, allowing for the quantitative determination of the selected alkaloids, namely berberine, coptisine, chelerythrine, chelidonine and sanguinarine (Figure S1). The mobile phase optimization was achieved by mixing DES with organic solvents and a novel approach to fine-tuning DES properties by mixing eutectic solvents of differing properties.

## 2. Results and Discussion

Deep eutectic solvents have attracted more and more attention within the last few years, which has resulted in a significant increase in the number of articles and, as a consequence, a broadening and deepening of the research field. The emerging papers bring new information regarding DES properties [17,18,19,20,21] and their applications [12,13,22,23] but also use a retrospective view in order to provide a categorization of the already gathered knowledge [6,8,18]. The latter group is particularly significant for the relatively young and rapidly growing research field of DES, as it allows for better understanding and thus improved methodological correctness of future investigations. The primary classifications are the distinction of hydrophilic and hydrophobic DES and the division of eutectic solvents into classes (I–V, to the date), which facilitates selecting optimal DES for chromatographic purposes. 

The presented research continues the preliminary studies on eutectic TLC [13]. In the current investigation, a similar working matrix was employed because it proved its suitability by combining the important features, such as the possibility of detection without derivatization, thermostability or being a mixture of significant complexity.

### 2.1. DES Selection and Preparation

Based on the previous results [13], the main focus was on the eutectic consisting of terpene compounds because only such ones previously enabled sufficient chromatographic efficiency regarding the investigated working solution. Recently, multiple new DESs based on terpenoids emerged. They can be found both in scientific papers [8,22,24] and in professional (mostly industry-orientated) databases [25]. However, none of those DESs was tested as a component of a TLC mobile phase up to date. In the experiment, the inclusion parameters for the primary DES were: at least one of the compounds should be a terpenoid, and the second component should be a nature-occurring molecule. Furthermore, the best performing of the previously investigated hydrophobic DESs were included (E_4_, E_9_, E_13_) [13]. All the selected primary DESs (E_1_–E_13_, Table 1) could be described as hydrophobic and belonging to the type-V eutectic solvents. Additionally, some choline chloride-based DESs (type-III, hydrophilic) were included as possible polar modifiers of the mobile phases (E_14_–E_17_). Considering that in the last experiment, the combination of choline chloride (ChCl) with sugars resulted in excessive viscosity, this time, organic acids and phenol were selected as a co-eutectics. A detailed explanation of other preferable parameters in selecting DES, such as low viscosity or stability during the chromatographic process is discussed in the previous article [13]. All the investigated DESs are gathered in Table 1.

Where possible, the molar ratio of eutectic mixtures was based on literature data. In the absence of such information, the default equimolar ratio was applied. The preferred obtaining procedure was P1, as it is considered to be the simplest and most sparing [8]; in the case of not forming the stable and homogenous liquid, P2 was applied. Solvent-mediated liquefaction (P3) was treated as the last resort. According to the literature, the differences in the obtaining method do not affect DES properties [26].

### 2.2. Chromatographic Analysis with Pure DES

In the first step, all the eutectic liquids were used purely as mobile phases in order to estimate their basal chromatographic properties. Similarly to the earlier report, none of the pure tested DES enabled carrying a satisfactory separation of the working standard solution. 

The average development time ranged from 3 to 6 h, except for the E_8_ phase, where the time was significantly shorter (70 min). On the other hand, the longest chromatographic process was observed for the E_7_ phase (>24 h). Interestingly, both the fastest and the slowest mobile phases included menthol. A similar phenomenon was also observed for the ChCl-based mobile phases (E_15_, E_16_, E_18_), where the inclusion of malic, oxalic or lactic acids was conditioning development times close to 24 h, while ChCl:phenol (E_16_) was significantly faster. For comparison, the E_5_ (menthol and AA) and E_6_ (menthol and borneol) DES developed for about 6 h. The number of hydroxyl groups with low steric hindrances correlates with significant viscosity. This is in line with the previously noted regularity that hydroxy acids (also sugars) grant high viscosity [13] as well as with the literature data, which estimate the viscosity of menthol:lactic acid DES as being over 25 times higher compared to menthol:AA DES [27]. It is also consistent with the theoretical basics of creating a eutectic matrix [27].

One of the significant factors limiting the development of the eutectic TLC was the scarcity of low-viscosity eutectic liquids, which translated into a long duration of a single analysis, thus limiting the method’s usefulness [13]. Generally, for the widely understood chromatographic purposes, lower expected viscosity is favorable as it is associated with lower back pressure in the case of HPLC systems or shorter time of plate development in TLC [6,13]. Therefore, when selecting the chromatographic mobile phases, the limited number of hydroxyl groups within the eutectic system may be perceived as beneficial. However, it should be noted that for the specific applications, researchers may prefer high-viscosity DES [9]. Furthermore, viscosity is not the sole parameter to be taken into account. However, in both cases, considering such group dependencies facilitates the selection of the optimal eutectic solvent for researchers from different scientific fields.

**Figure 1:**
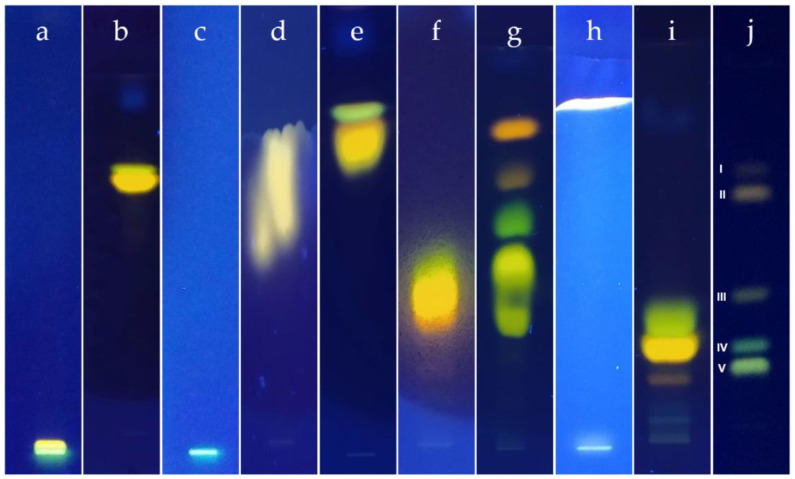
Chromatograms of the selected chromatographic systems (Si60 plates, unless otherwise noted; 366 nm). Mobile phases, left to right: (**a**) E_12_; (**b**) E_11_; (**c**) E_5_; (**d**) E_15_; (**e**) E_11_-A5; (**f**) E_11_-C30; (**g**) E_3_-M20; (**h**) EM_10_; (**i**) EM_6_; (**j**) EM_3_-M20 (HPTLC). Alkaloids: I—chelidonine, II—sanguinarine, III—chelerythrine, IV—berberine, V—coptisine.

Most of the E_1_–E_13_ mobile phases did not significantly move investigated compounds from the start line and almost did not separate the mixture—the investigated alkaloids did not exceed R_f_ 0.2 (Figure 1a). The exceptions were DESs containing acids, such as E_2_, E_7_, or particularly E_11_ composed of thymol and AA (Figure 1b), which allowed a certain degree of separation but with a suboptimal development time of 3.5 h. Interestingly, menthol coupled with AA in the same molar ratio was not polar at all (Figure 1c), which suggests that in that case, it is not AA that is responsible for the polarity but the saturation of the terpene-molecule instead. On the other hand, according to the initial assumptions, the ChCl-based eutectic solvents moved the investigated compounds towards the solvent front (Figure 1d). However, it should be noted that they were included in the experiment only as potential modifiers of the mobile phases. Their analysis was carried out to estimate their basal properties as the subsequent additives.

### 2.3. Chromatographic Analysis of Diluted DES

As proven in the previous article, the dilution of pure DES with various solvents was an effective way of modifying DES elution strength and viscosity [13]. The hitherto published results were, however, suboptimal for densitometric analysis. Similar diluting organic solvents were used to continue the research direction, with minor changes. Water was excluded from the experiment because it did not mix with the hydrophobic DESs selected as the primary mobile phases. In turn, AA was included based on already gathered results. Analogously to the earlier research, the dilution steps were projected to not exceed 40% to avoid breaking the eutectic matrix [10,21].

In the current experiment, the greatest emphasis was placed on DES, which has not been analyzed in TLC so far (E_1_–E_3_, E_5_–E_8_, E_10_–E_12_, Table 1). The chromatographic properties of the diluted DESs were investigated similarly to the pattern presented in the previous article [13], briefly, by mixing 10, 20 or 30% of the organic solvent with the individual DES, with analysis in an ascending manner. The 40% dilution was initially planned, but sufficient migration was achieved with a lower percentage in every case. All the analyzed DESs were miscible with acetone, ethyl acetate, diethyl ether and chloroform and methanol. Contrary to the earlier experiment, the latter solvent was also mixed with E_4_ DES. The issue has been thoroughly analyzed: the triple repetitions performed similarly. After referring to the notes and protocols from the previous part of the experiment, the only tracked difference was limited to the source of the chemicals used, which suggests the impurities are responsible for the phenomenon. That is in accordance with literature data, where great emphasis is placed on the purity of DES components and reproducibility resulting therefrom [8].

Methanol and acetone were performing the best as co-eutectic mobile phase components in the described experiment, considering the solvents employed. They generally maintained forming recognizable bands visible in the chromatography with pure DES and improved resolution. Thus, they were treated as the main diluting agents in the further steps. The initial effort regarding optimization was put on the E_11_ DES, which enabled the best results for the pure DES. To improve the results, the addition of 5% methanol or acetone (E_11_-M5 and E_11_-A5, respectively; Figure 1e) was tested, but in either case, the addition caused moving all the alkaloids to the front line. Based on the information from the earlier experiments [13], chloroform was employed as the solvent without significant impact on the polarity yet was able to decrease the time of development. Indeed, the E_11_-C30 phase was 30% faster than the pure DES, but still, the time was unsatisfactory (over 2 h). Moreover compared to the initial DES bands, they were blurred, which deteriorated the resolution (Figure 1f). Therefore, the mobile phase was no longer investigated as single.

The DESs previously not employed in TLC were investigated after dilution with methanol or acetone in the next step. Most of them allowed partial separation within the range of 15–30% addition of the solvents (Figure 1g). However, none of the cases demonstrated that the chromatograms were significantly better than the previous results [13]. Dilution of selected DESs (E_3_, E_7_, E_9_) with AA (10% or 20%) significantly lowered the R_f_ values, contrary to what was expected. Moreover, for E_9_, DES addition of AA significantly increased the development time. Explaining the nature of the above phenomenon needs additional research, which is not provided within the presented study.

### 2.4. Chromatographic Analysis of DES’s Mixtures

Given that none of the dilutions of a single DES with standard solvents within the projected experiment enabled the chromatographic separation of sufficient quality (allowing the densitometric analysis), a different approach was needed. Dilution with solvents turned out to have its restrictions, from which limited regulation capacity is one of the most important. Therefore, it was a matter of question if any other DES can be treated as polarity modifiers for eutectic liquids, apart from standard solvents. The preliminary results showed that eutectic solvents are not freely miscible within the group. Terpene-based DESs were miscible with each other in every investigated case. On the other hand, ChCl-based DESs, when mixed with terpene-based ones, did not give obvious results and simple conclusions. The preliminary test results involving the mixing of selected eutectic liquids are presented in Table 2.

It is noteworthy that the results show that the mutual miscibility of terpene- and ChCl-based DESs is not a simple hydrophilic/hydrophobic-properties matter. Moreover, in the investigated phenomenon, even differences in belonging to eutectic classes (III for chloride-based DESs and V for terpene-based ones) were not an exclusion factor. The issue is intriguing and needs additional in-depth research as it may have possible implications for understanding eutectic matrix functioning.

The mixtures of DESs were created based on the results obtained: miscible liquids strongly differing in chromatographic performance were paired. To improve readability, the newly created eutectic mixtures were given separate names (Table 3).

**Figure 2:**
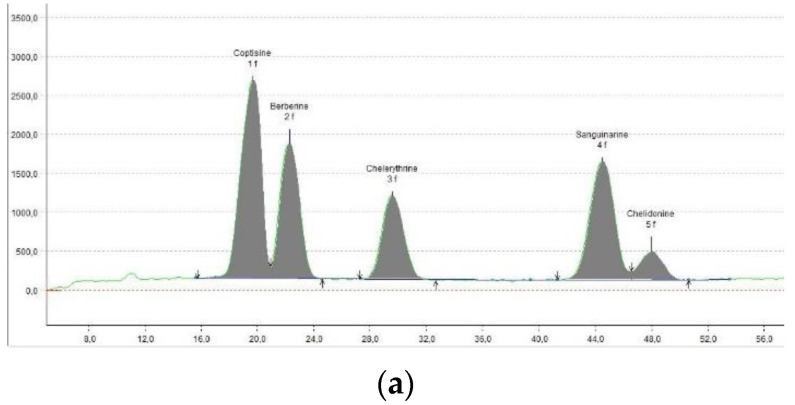

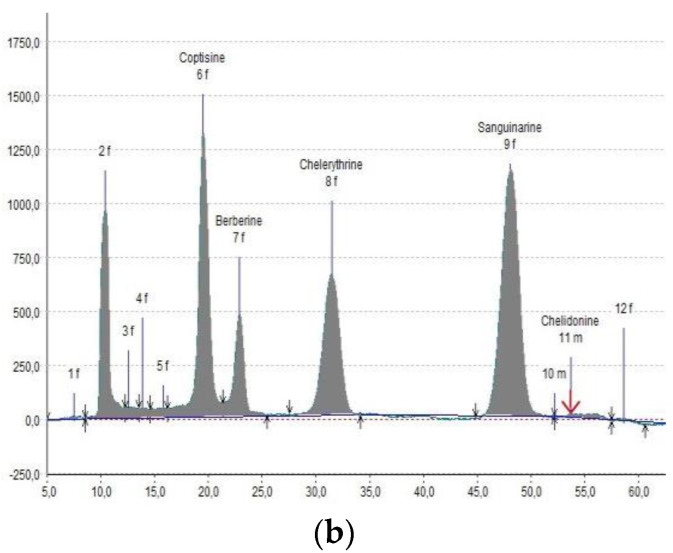
Densitograms of: (**a**) working mixture of standards; (**b**) real sample. Densitograms obtained at 340 nm using HPTLC Lichrospher plates and EM_3_-M20 mobile phase.

The initial default ratio was 1:1, and in the case of promising results, the additional ratio values were also tested. Mixed DESs were investigated both as pure mixtures and after dilutions. Pure mixtures were differing in their polarity. The perspective results were obtained with the EM_2_ mix, so additional ratios (EM_3_–EM_5_) were also included. The mixes containing ChCl (EM_7_–EM_10_) did not grant the expected results because no satisfactory bands were formed in any of the cases (Figure 1h). Therefore, they were excluded from further investigation. In the next step, the best performing mixtures were diluted with the solvents, analogously to the pure DESs (Section 2.3). Given that the mixtures had increased polarity compared to the best performing DESs, the addition of solvents typically did not exceed 20%. Generally, methanol performed as the best diluting solvent in all the investigated cases. When diluted with methanol, the mixes EM_2_–EM_5_, all derived from E_3_ and E_8_ DESs, gave the most interesting results. After optimization, the EM_3_-M20 mobile phase gave separation of densitometric quality. The result was repeated on HPLTC plates, with further resolution improvement, and confirmed on a real sample (Figure 1j and Figure 2). The development time was only 50 min.

The obtained results suggest that during eutectic TLC optimization, it is more practical to start with a DES of relatively low polarity and adjust the parameter up with a polar solvent. This approach allows better regulation of an elution strength and a concentration-dependent decrease in the development time of the resulting mobile phase, compared to the pure DES.

During the optimization procedure, it was observed that the visualization of bands could be substantially improved by heating the developed plates. NADES are generally perceived as non-volatile liquids, and the information is repeated in numerous articles ([18,28,29]—to name only a few). Most likely, the claim is valid mainly for the sugar-, sugar alcohol- and amino acid-based DES. Given that terpenes are generally volatile, it was expected that DESs composed thereof might also share the feature. The assumption was supported by the observation that terpene-based DESs are fragrant. The literature data confirms the volatility of the mentioned eutectic liquids [27]. Therefore, it was checked if heating could remove mobile phase leftovers from the chromatographic plate. The results showed that heating over 100 °C allows at least partial evaporation of the mobile phase, but the best results were achieved by heating the plate at 150 °C for 5 min. After the procedure, the plates were “dry” and practically odorless. However, traces of mobile phase leftovers were still detectable at 254 nm due to the fluorescence indicator in plates, even when prolonged time (up to 2 h) or increased temperature (up to 200 °C) was used. That can be considered a limitation in the use of eutectic TLC. However, at 366 nm, no interferences were visually observed. Based on the results presented above, an additional attempt was made to spray-derivatize the heated plate with the Draggendorf’s reagent to determine if spray-derivatization is possible in eutectic TLC. The procedure was successful; orange bands were formed as expected, but the derivatization was not included in the final protocol. The optimized system underwent validation according to the ICH guidelines [30]. Validation details are presented in the supplementary material (Table S1). The obtained results draw attention to the linearity range, which starts from 1 ng of pure compound per band. This value is many times lower than the already-existing classic TLC method for quantification of the investigated alkaloids [31]. 

The optimized method was subsequently tested with the real sample obtained from the *Chelidonium maius* herb and proved to be efficient. The content of the five investigated alkaloids is presented in Table 4.

The amount of the investigated alkaloids is comparable to the results found in the literature data [32], which provides additional confirmation that eutectic TLC is a valuable alternative to conventional TLC.

In the preliminary results, the development time was limiting the usefulness of the eutectic TLC—in the case of a single analysis, times reaching two hours were not acceptable for a routine procedure [13]. However, the issue was successfully overcome by choosing optimal DES selected from a growing number of possibilities, just as predicted in the previous article. Thus, one of the most serious limitations of the method was avoided. Moreover, post-development heating of plates allowed for minimization of the plate impregnation effect by mobile phase leftovers in the case of terpene-based DESs. That, in turn, enabled attempts of spray-derivatization, which further increases the functionality of eutectic TLC. From what it seems now, eutectic chromatography would improve separation possibilities compared to traditional TLC due to additional interactions with a eutectic matrix. It suggests that the eutectic TLC may be a good choice for difficult samples compared to already existing complicated approaches, such as multigradient or 2D TLC. Furthermore, eutectic solvents as mobile phases do not require high investment because creating a DES in most cases is extremely simple (requires just mixing or/and heating). Thus, eutectic TLC gives new separation opportunities without creating an exclusionary equipment barrier.

## 3. Materials and Methods

### 3.1. Chemicals

All of the solvents and reagents used in the experiment were of analytical grade. Choline chloride, citric acid monohydrate, DL-camphor, thymol, L-menthol, phenyl salicylate and chloral hydrate were obtained from Merck (Merck, Darmstadt, Germany), acetone, chloroform, diethyl ether, methanol and other chemicals were purchased from POCh (Poch, Gliwice, Poland). 

### 3.2. Standards and Sample

Berberine, coptisine, chelerythrine and chelidonine were obtained from Sigma Aldrich (Sigma Aldrich, St. Louis, MO, USA), and sanguinarine was obtained from Extrasynthese (Extrasynthese, Genay, France). Stock solutions (0.1 mg mL^−1^) were prepared by dissolving the compounds (1 mg) in 10 mL of methanol, while the working standard solution was obtained by mixing an equal amount of each standard solution in a vial and a 100-fold dilution. The real sample was prepared from *Chelidonium maius* L. herb, analogously to the procedure described in the previous paper [13,32]. Briefly, 0.1 g of dried plant material was extracted twice with 10 mL of methanol with 0.05 M of hydrochloric acid in an ultrasonic bath for 15 min. The extracts were combined, evaporated to dryness and dissolved in 10 mL of methanol.

All the solutions were kept at –18 °C prior to analysis.

### 3.3. Mobile Phases Preparation

The eutectic mobile phases were prepared analogously to the procedures described in the previous paper [13]. Briefly, procedure 1 (P1) was a spontaneous liquefying of the combined compounds. Procedure 2 (P2) involved mixing the components and subsequent heating up to 80 °C until a homogenous and stable liquid was formed. Procedure 3 (P3) was modified compared to the previous research and required the addition of a mixture of methanol and water (3:1, *v*/*v*) until all the compounds were dissolved, and subsequent evaporation of the diluents at 60 °C under reduced pressure using a rotary evaporator (R-210, Büchi, Germany), to the stable mass. Eutectic solvents included in the experiment are listed in Table 1. 

Similarly to the earlier work [13], the eutectic liquids were used pure or diluted with up to 30% of acetic acid, methanol, chloroform, diethyl ether, or acetone (AA, M, C, E or A, respectively), e.g., E_1_-M10 means that DES named E_1_ contains 10% of methanol (wt/wt).

During the optimization stage, the fine adjusting of mobile phases’ properties was carried out either by adding a mixture of organic solvents to a single DES or by combining two different eutectic solvents with the possible subsequent addition of organic solvents to the mixture. In the first case, mobile phases were prepared by diluting a single DES (wt/wt) with a pre-prepared mixture of two organic solvents (1:1, *v*/*v*). In the second case, the selected DESs were investigated as an equiweight ratio mixture of two eutectic liquids (wt/wt) with an appropriate addition of a relevant solvent, prepared as described above. All the modifications are presented in Table 3.

Miscibility of DESs was investigated by mixing equal amounts of both analyzed liquids in an Eppendorf tube and subsequent vigorous shaking for 30 s. If a homogenous, clear, and translucent liquid was formed, DESs were considered miscible. If any cloudiness or border between liquids was observed, DESs were considered immiscible (Table 2).

### 3.4. Equipment and Chromatographic Conditions

Planar chromatography was performed on the TLC Si60 and HPTLC Lichrospher Si60 (both glass support, with fluorescence indicator, 10 × 20 cm; MERCK, Merck, Darmstadt, Germany) according to the procedures described in the previous paper [13]. Briefly, the plates were pre-washed with a mixture of chloroform and methanol (1:1). Next, the standards or samples were applied to the plates using a Biostep-Desaga AS 30 autosampler as 5 mm bands. Chromatography was carried in horizontal Teflon DS-chambers (Chromdes, Lublin, Poland). The plates were developed for 7 cm and dried for 5 min at 150 °C on a hot plate (Thermoplate S_plus_, Biostep-Desaga) and analyzed in UV light (366 nm). The selected chromatographic system was subjected to the densitometric analysis using a CD 60 densitometer (Biostep-Desaga, Wiesloch, Germany) and the Desaga Proquant software (Ver. 3.05; Biostep-Desaga, Wiesloch, Germany). The working wavelength (λ = 340 nm) was selected after in-situ analysis of standards, using a mercury lamp and a fluorescence mode. The slit dimensions were 0.02 × 6 mm (height and width, respectively). 

Derivatization was performed by spraying the developed plates with Draggendorf’s reagent with Camag Derivatizer (Camag, Muttenz, Switzerland), using the reagent prepared according to the equipment manual.

All the experiments were performed in ambient conditions.

### 3.5. Validation

The optimized chromatographic system was validated according to the International Conference on Harmonization (ICH) guidelines [30], regarding specificity, linearity, accuracy, limits of detection (LOD), limits of quantification (LOQ), repeatability and intermediate precision, with the detailed procedure according to the protocol presented in the previous paper [33]. The additional specifications and results of the validation are presented in the supplementary materials (Table S1).

## 4. Conclusions

The presented results, describing the first TLC system allowing for qualitative and quantitative analysis with a DES-based mobile phase, withdraw the eutectic TLC from an early gestation stage. The comparison with the reference results obtained with conventional TLC [31] shows the full usefulness of the new method. In the next step, broadened research is needed, including the separation of compounds other than alkaloids. Moreover, the range of the investigated DES should be increased over the hereby presented hydrophobic, natural-based, type V eutectic solvents. It would allow for assigning the range of default DESs; alternatively, the suggested properties are optimal for chromatographic analysis of the specific group of compounds, similar to the generally accepted manuals [34].

Nevertheless, given the results, eutectic TLC may be from now treated as an applicable and operational method.

## Figures and Tables

**Table 1:** List of pure DES.

Components	Molar Ratio	No	Obtaining Procedure
borneol + phenol	1:1	E_1_	P2
camphor + formic acid	1:1	E_2_	P1
camphor + phenol	1:1	E_3_	P1
camphor + phenyl salicylate	1:1	E_4_	P1
menthol + acetic acid	1:1	E_5_	P1
menthol + borneol	8:2	E_6_	P2
menthol + lactic acid	1:2	E_7_	P1
menthol + limonene	1:1	E_8_	P1
menthol + phenol	1:1	E_9_	P1
menthol + thymol	1:1	E_10_	P1
thymol + acetic acid	1:1	E_11_	P2
thymol + linalool	1:1	E_12_	P1
thymol + phenol	1:1	E_13_	P1
choline chloride + malic acid ^1^	1:1	E_14_	P3
choline chloride + oxalic acid ^1^	1:1	E_15_	P2
choline chloride + phenol ^1^	1:2	E_16_	P1
choline chloride + lactic acid ^1^	1:1	E_17_	P3

^1^ DES intended as modifiers.

**Table 2:** Mutual miscibility of terpene- and ChCl-based DESs (E_1_–E_13_ and E_14_–E_17_, respectively).

	E_1_	E_2_	E_3_	E_4_	E_5_	E_6_	E_7_	E_8_	E_9_	E_10_	E_11_	E_12_	E_13_
E_14_	−	*	−	−	−	−	−	−	−	−	−	−	−
E_15_	*	*	−	−	−	−	−	−	−	−	−	−	−
E_16_	*	+	+	+	+	+	+	−	+	+	+	+	+
E_17_	−	−	*	−	−	−	−	−	−	−	+	−	+

(−)—not miscible; (+)—miscible; (*) solidified.

**Table 3:** List of mixed DESs.

DES Mix Name	Component 1	Component 2	Component 1 to 2 Ratio
EM_1_	E_3_	E_11_	1:1
EM_2_	E_3_	E_8_	1:1
EM_3_	E_3_	E_8_	2:1
EM_4_	E_3_	E_8_	3:1
EM_5_	E_3_	E_8_	3:2
EM_6_	E_9_	E_11_	1:1
EM_7_	E_17_	E_11_	1:1
EM_8_	E_17_	E_13_	1:1
EM_9_	E_16_	E_2_	1:1
EM_9_	E_16_	E_11_	1:1
EM_10_	E_16_	E_13_	1:1

**Table 4:** Quantitative analysis of dried plant material in terms of the investigated alkaloids (berberine, chelerythrine, chelidonine, coptisine, sanguinarine) expressed as mg g^−1^ dry weight.

Compound	Concentration (mg g^−1^ Dry Weight)
Berberine	0.636 ± 0.052
Chelerythrine	0.789 ± 0.067
Chelidonine	0.216 ± 0.035
Coptisine	0.992 ± 0.013
Sanguinarine	1.410 ± 0.009

Mean value ± SD, *n* = 3.

## Data Availability

The data presented in this study are available in supplementary material.

## Supplementary Materials

The following supporting information can be downloaded at: https://www.mdpi.com/article/10.3390/molecules27092960/s1, Figure S1: The chemical structures of the investigated alkaloids. Be—berberine, Ce—chelerythrine, Ci—chelidonine, Co—coptisine, S—sanguinarine; Table S1: Validation data for the optimized method (*n* = 6).

## Sample Availability

Samples of the compounds are available from the authors.
