# Supplementary material for: Eutectic Thin-Layer Chromatography as a New Possibility for Quantification of Plant Extracts—A Case Study
Source: Molecules. 2022 May 5;27(9):2960. doi: 10.3390/molecules27092960 (PMC9105703; doi:10.3390/molecules27092960)
Supplement: Supplementary file 1 [file molecules-27-02960-s001.zip › molecules-1685117-supplementary.pdf]

QUANTITATIVE ANALYSIS OF CHELIDONIUM ALKALOIDS USING EUTECTIC THIN-LAYER CHROMATOGRAPHY

Danuta Raj

Department of Pharmacognosy and Herbal Medicines, Wroclaw Medical University, Borowska 211a, 50-556 Wrocław, Poland; danuta.raj@umw.edu.pl

Supplementary Materials

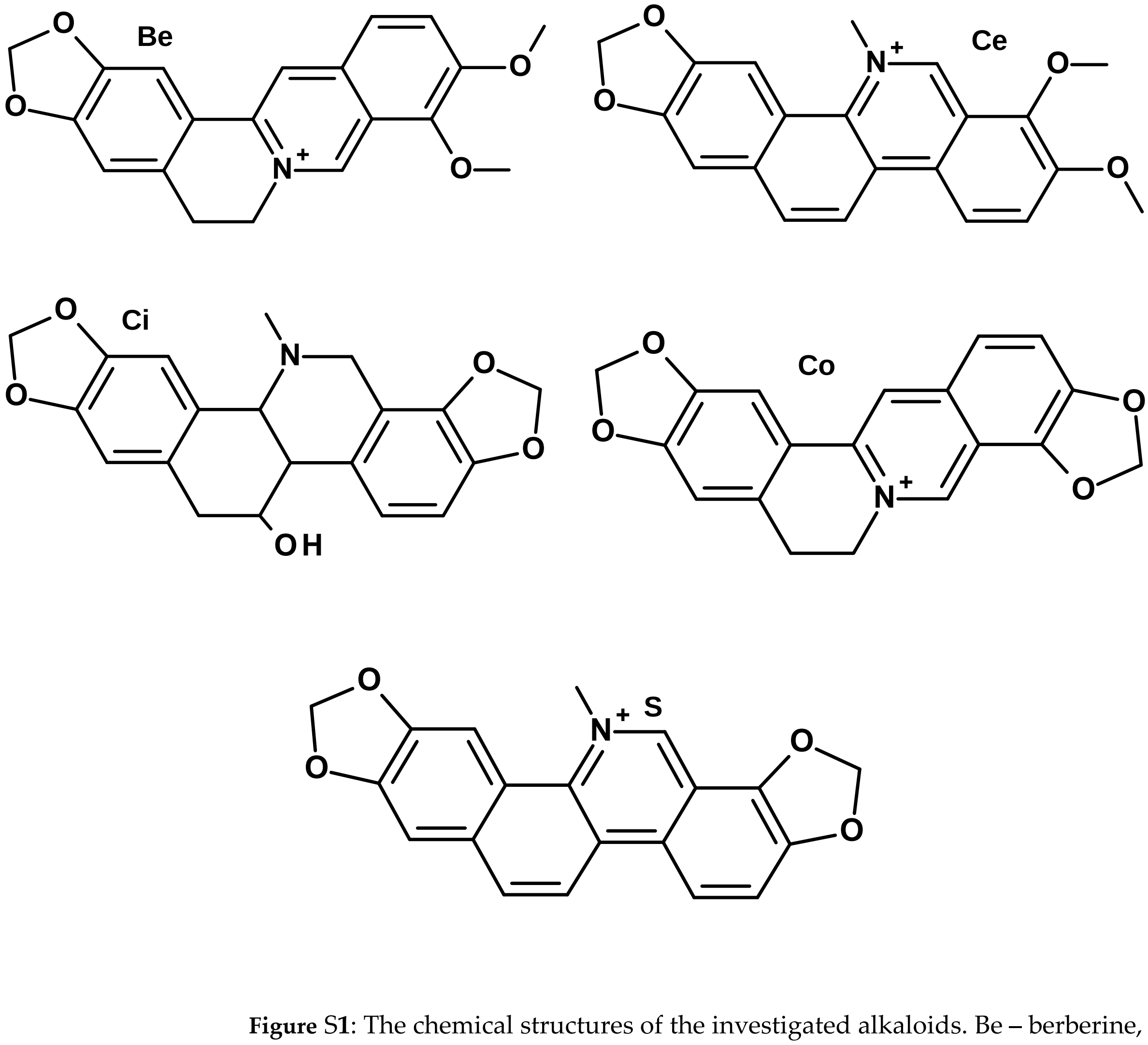

Figure S1: The chemical structures of the investigated alkaloids. Be – berberine, Ce – chelerythrine, Ci – chelidonine, Co – coptisine, S - sanguinarine

Validation

The validation of the developed method (Tab. S1) was performed using all the investigated alkaloids, namely berberine, chelerythrine, chelidonine, coptisine and sanguinarine, according to the International Conference on Harmonization (ICH) guidelines [30]. For specificity, compounds were identified on the basis of R_f values compared to the standards, UV spectra and color of the bands before and after derivatization. Linearity was determined by constructing calibration plots of all the standards, on the basis of peak areas using eight different calibration samples (1-100 ng per band), n=6. Limits of detection (LOD) and quantitation (LOQ) were calculated from AG and AA calibration equations as the amounts for which the signal-to-noise ratios (S/N) were ≥3:1 and ≥10:1, respectively, with use of a blank sample. Repeatability was calculated by applying the investigated alkaloids in the amount of 20, 40 and 60 ng per band. The same sample was independently analyzed seven times on each HPTLC plate. Intermediate precision was measured by analysis of seven samples (40 ng per band), prepared with the same procedure, and analyzed with the same chromatographic conditions (on the same plate) on one day (intra-day precision) and on three different days (inter-day precision). As for the accuracy, recovery was measured for standards added, at three concentrations, to 1 mL extract solution of known composition (n = 6).

Table S1. Validation data for the optimized method (n = 6).

Standard	Berberine	Chelerythrine	Chelidonine	Coptisine	Sanguinarine
R_f	0.32	0.43	0.69	0.28	0.64
Calibration equation	y=458.2084x+2750.6101	y=587.0374x+1101.0571	y=159.7340x+397.3515	y=1495.1971x+1775.8018	y=701.2964+1993.0061
Correlation coefficient (r^2)	0.99276	0.99685	0.99403	0.99879	0.99532
Linear range [ng]	1 -100	1 -100	1 -100	1 -100	1 -100
LOD [ng]	0.14	0.23	0.08	0.31	0.25
LOQ [ng]	0.52	0.61	0.46	0.75	0.67
Repeatability (CV, [%])a)					
20 ng	0.28	0.17	0.08	0.10	0.21
40 ng	0.42	0.47	0.22	0.17	0.35
60 ng	0.31	0.62	0.27	0.48	0.36
Inter-day precision (CV, [%])a)	2.12	1.61	0.89	1.19	1.11
Intra-day precision (CV, [%])a)	3.37	3.55	2.29	3.01	3.32
Recovery [%] ± SD	101.49 ± 6.52	96.83 ± 2.27	104.21 ± 3.31	102.66 ± 2.11	98.72 ± 5.38

a) n = 7
